# Patient experiences of psychological therapy for depression: a qualitative metasynthesis

**DOI:** 10.1186/s12888-020-02682-1

**Published:** 2020-06-18

**Authors:** Susan McPherson, Claire Wicks, Ilaria Tercelli

**Affiliations:** School of Health and Social Care, University of Essex, Colchester, CO4 3SQ UK

**Keywords:** Depression, Psychological therapies, Patient experience, Patient choice, Guidelines

## Abstract

**Background:**

Globally, national guidelines for depression have prioritised evidence from randomised controlled trials and quantitative meta-analyses, omitting qualitative research concerning patient experience of treatments. A review of patient experience research can provide a comprehensive overview of this important form of evidence and thus enable the voices and subjectivities of those affected by depression to have an impact on the treatments and services they are offered. This review aims to seek a comprehensive understanding of patient experiences of psychological therapies for depression using a systematic and rigorous approach to review and synthesis of qualitative research.

**Method:**

PsychINFO, PsychARTICLES, MEDLINE, and CINAHL were searched for published articles using a qualitative approach to examine experiences of psychological therapies for depression. All types of psychological therapy were included irrespective of model or modes of delivery (e.g. remote or in person; group or individual). Each article was assessed following guidance provided by the Critical Appraisal Skill Programme tool. Articles were entered in full into NVIVO and themes were extracted and synthesized following inductive thematic analysis.

**Results:**

Thirty-seven studies, representing 671 patients were included. Three main themes are described; the role of therapy features and setting; therapy processes and how they impact on outcomes; and therapy outcomes (benefits and limitations). Subthemes are described within these themes and include discussion of what works and what’s unhelpful; issues integrating therapy with real life; patient preferences and individual difference; challenges of undertaking therapy; influence of the therapist; benefits of therapy; limits of therapy and what happens when therapy ends.

**Conclusions:**

Findings point to the importance of common factors in psychotherapies; highlight the need to assess negative outcomes; and indicate the need for patients to be more involved in discussions and decisions about therapy, including tailoring therapy to individual needs and taking social and cultural contexts into account.

## Background

In the United Kingdom (UK), United States (US) and other developed economies, evidence based treatment guidelines can influence the varieties of treatments made available to people formally diagnosed with depression, including the UK National Institute for Health and Care Excellence (NICE) guideline on depression [1]; the American Psychiatric Association guideline [2]; the European Psychiatric Association (EPA) guideline on psychotherapies for chronic forms of depression [3]; and the more recent American Psychological Association depression guideline [4]. All of these guidelines prioritise Randomised Controlled Trials (RCTs) and quantitative meta-analyses of trials as might be expected, since this is a key feature of the evidence based medicine paradigm. Yet there are also different emphases within these guidelines, reflecting the contested nature of evidence and interpretation within the paradigm.

As a result of differences in approach to trial evidence and synthesis and the ways that guideline committees are formed and operate, variations appear in terms of which psychological treatments are recommended for depression in adults and whether these are recommended as a range of choices or as a hierarchical list. For example, EPA recommends, for persistent depression, Cognitive Behavioural Analysis System of Psychotherapy and Interpersonal Therapy (IPT) as first and second line treatments with Cognitive Behavioural Therapy (CBT), Psychodynamic Psychotherapy (PP), Problem Solving Therapy (PST), Schema Therapy, Radical Openness Dialectical Behaviour Therapy and Mindfulness Based Cognitive Therapy (MBCT) as third line treatments; whereas the American Psychological Association recommends Behavioural Therapy, Cognitive Therapy, CBT, MBCT, IPT, PP or supportive therapy (with no particular hierarchy).

Since 2008, the UK has established a national psychological therapy service which has provided NICE recommended therapies for depression and anxiety. Over 900,000 people access these services every year, and the National Health Service aims to increase this figure to at least 1.5m by 2021 [5]. Although initially only providing CBT (following the hierarchy of recommendations in the 2004 NICE depression guideline), therapies available in these services have expanded slightly in some areas to other ‘evidence based’ therapies as determined by NICE updates. NICE depression guidelines to date have been significantly more complex than other guidelines in terms of the sub-classification of depression, branding of therapies and hierarchical ordering of treatments and prescription of specific combinations.

Although many guidelines refer to the importance of patient choice and experience, none of them include a review of qualitative research concerning patient experience of treatments. It has been noted that a review of patient experience research is critical in order to enable the voices and subjectivities of those affected by depression to have an impact on the treatments and services they are offered, in keeping with a drive for a more patient-centred health service [6]. Such a review could usefully inform guidelines as well as psychological treatment approaches in practice, since service user preferences for particular psychological approaches are known to be associated with better outcomes, a sense of fulfilment and fewer dropouts [7]. RCTs are relatively poorly equipped as a methodology to provide sufficient detail on process, context and individual differences that might support patients to make more informed choices.

When presented with findings of an RCT of psychological therapy for depression, service users and carers reported that the findings were of limited value in enabling informed choice since it failed to reveal the complex processes in therapy that often depended on unique therapist and patient variables [8]. A review and synthesis of qualitative studies concerning patient experiences of psychotherapies could therefore provide a useful source of information for patients and primary care professionals discussing individual treatment options which take social and individual factors into account. It has also been noted that RCTs of psychological therapies often fail to collect sufficient data on adverse effects [9] partly owing to an assumption that talking therapies have limited potential for harm. Given the lack of systematic monitoring of adverse outcomes and that RCTs consistently use researcher selected (as opposed to patient-preferred) outcome measures, a review of qualitative research could identify whether there are any specific types of harm or benefits that might be important to monitor in future research or address in treatment manuals and practitioner training.

Qualitative research is increasingly used in health service research to understand subjective experiences of conditions and treatments and has been increasingly used in psychotherapy research. There are a range of approaches to synthesising sets of qualitative studies deriving from different disciplines, such as meta-ethnography (from anthropology) or ‘formal grounded theory’ from sociology [10]; a common aim is to generate an overall comprehensive synthesis of the available individual qualitative studies on the topic. Approaches to metasynthesis vary in the way in which data is synthesised and analysed, but tend to be underpinned by the notion of systematically searching for and identifying primary qualitative research on the same topic and abstracting the findings to a ‘meta’ level. Qualitative metasynthesis is related to, but not identical to secondary data analysis, in that the primary data sets of each study are not available to reviewers. In this sense, qualitative metasynthesis is limited in terms of its relative distance from the first person raw material, relying on author interpretations of primary data. While some approaches to qualitative metasynthesis make use of the selected raw data within primary research reports (e.g. [11]), this could lead to bias. Sandelowski and Barroso have argued that the ‘data’ within a metasynthesis should therefore consist of study findings (author interpretations) rather than raw data [12]; the latter approach has been applied in the current review.

The aim of the present review is to synthesise existing qualitative evidence concerning patient experiences of psychological treatments for depression with a view to improving informed choice and informed consent to psychological treatment. No comprehensive review of this body of literature has been carried out to date. The 2009 NICE guideline included a chapter on patient experiences; however, this focused on experiences of depression, management and coping, rather than experiences of treatments. The chapter included a secondary analysis of 38 ‘healtalkonline’ accounts but with very limited analysis concerning psychosocial treatments (p88–90). The chapter also presented a narrative review of selective literature: a systematic review of nine studies about experiences of self-help in primary care [13]; a study of experiences of depression based on the same 38 healthtalkonline accounts [14]; and a further study on patient experiences of primary care [15]. In the Khan et al. [13] review, one of the 9 studies [16] looked at experiences of therapy (and is included in the present review). Therefore, although this chapter in the NICE guideline acknowledges aspects of patient experience, it does not constitute a review of patient experiences of therapy which could inform treatment recommendations and patient choice.

A previous qualitative metasynthesis reviewed eight studies of patient experiences of computerised CBT for depression or anxiety [11]. The scope differed to the current review in that it included adolescents as well as adults and included people with anxiety disorders. Two of the studies reviewed are included in the present review. However, given the narrow focus on the single delivery mode, there remain questions about what the broader body of literature on patient experiences of psychological treatments for depression might reveal about the overall landscape of psychological therapy provision.

### Objectives

The current review aimed to seek a comprehensive understanding of patient experiences of psychological therapies for depression across all modalities, using a systematic and rigorous approach [10]. Studies included participants who were adults with depression who had received psychological treatment for depression. Studies were qualitative and had explored patient experiences using qualitative interviews or focus groups.

## Methods

### Data sources

An electronic search was conducted in June 2019 using the databases PsychINFO, PsychARTICLES, MEDLINE, and CINAHL.

### Search strategy

The search terms were chosen with a view to identifying studies concerned with depression using a qualitative approach and focusing on experiences of psychological therapies (broadly defined). Some specific therapies were included as search terms to ensure the most common therapies recommended in guidelines were picked up in the search. However, generic terms for therapy were also included to pick up other brands of therapy or therapies sharing features of branded therapies without using the common brand names. The following search terms were used:

1. depress* (in Title) AND
2. (Interview* OR case stud* OR observ* OR view* OR experience* OR attitude OR belie* OR feel* OR perce* OR understand* OR opinion* OR interpret* OR “ethnograph*” OR qualitative OR phenomenolog* OR “grounded theory” OR “purposive sampl*” OR “content analysis” OR “thematic analysis” OR “constant compar*” OR “field stud*” OR “theoretical sampl*” OR “discourse analy*” OR “focus group*” OR hermeneutic*OR heidegger* OR colaizzi* OR husserl* OR “narrative analy*” OR “mixed methods”) (in Abstract) AND
3. (therap* OR psychotherap* OR CBT OR “cognitive behav#ral therapy” OR “Behav#ral Activation” OR “Interpersonal therapy” OR IPT OR “Short-term psychodynamic therapy” OR “Behav#ral Couples therapy” OR “Mindfulness-based cognitive therapy” OR MBCT OR “Cognitive behav#ral treatment”) (in Abstract)

### Study selection

Studies were limited to adult, human, English, peer reviewed, academic journal articles only with no date restriction. Screening of titles and abstracts was shared between authors to identify articles likely to be eligible. Full texts of identified studies were reviewed in full to check whether they met the inclusion criteria. Reference lists of eligible articles were searched to identify any additional studies; these were reviewed in full to check for eligibility. The 2009 NICE guideline patient experience chapter and a metasynthesis of experiences of computerised CBT [11] were examined to identify any additional eligible studies.

### Eligibility criteria

Studies were included in which participants were all adults (18+) (studies of adolescents and children were excluded). Participants had to have had depression as a primary diagnosis (bipolar disorder and postnatal depression were excluded and studies where a physical condition or dependency was the primary presentation were also excluded). All studies reported a formal mechanism by which depression was defined for purposes of participant eligibility (studies were not included if participants had self-diagnosed depression). Therefore, studies were required to have employed a diagnostic interview using the Diagnostic and Statistical Manual for Mental Disorders; or to have used a self-report measure that maps onto diagnostic criteria such as the Beck Depression Inventory or Patient Health Questionnaire; or participants had to have been referred for depression treatment by a medical professional. Studies had to report themes concerning the impact of psychological therapy on the participants. Studies had to elicit accounts from participants about their experiences, for example by interviews or focus group (studies only using recorded session material as data were excluded, as were case studies). Studies had to concern experiences of a psychological therapy (studies about psychoeducation only were excluded); therapy could be face-to-face or remote, with a therapist or computerised. Studies were included if some or all participants were known to be taking antidepressant medication; however, the focus of the study had to be on patient experiences of psychological therapy. Participants should have experienced the therapy (studies were excluded where participants were asked about hypothetical ideas of therapy).

### Quality appraisal

Each article was assessed following guidance provided by the Critical Appraisal Skill Programme tool [17] which provides prompts to consider of the quality of each article [18]. The tool is not used to provide an absolute score of quality but facilitates consideration of clarity of aims, appropriateness of methods, design and recruitment methods, suitability of data collection, researcher reflexivity, ethics, analytic rigour and clarity of findings.

### Data extraction and synthesis

Key information was extracted from each article concerning participants and methods. The variables of interest were discussed and agreed by all authors and set out in an Excel spreadsheet into which details for each study were extracted by one author and checked by another (see Tables 1, 2 and 3). All articles were then entered into NVIVO for analysis. Following the principles of inductive thematic analysis [55], the first and second authors read articles in full, allocating codes to salient features of the data. Following Sandelowski and Barroso [12], content coded were study *findings*; raw data from study participants were not included in the coding process. “Findings consist of the databased and integrated discoveries, judgments, and/or pronouncements researchers offer about the phenomena, events, or cases under investigation. Findings are researchers’ interpretations of the data they collected or generated in their studies.” [12] p142. The use of author interpretations only as data for this metasynthesis avoids potential bias which could arise from attempting to re-analyse primary data selectively presented as participant quotes in the original studies.

**Table 1:** Study characteristics (CB approaches)

Study	Therapy	Sessions (n x duration + number of follow-ups)	Data collection (time after end-of-treatment)	Country	Age (range or mean)	Sample size	% female	Key demographics (where available)
Allen et al. (2009) [19]	FtF; Group; MBCT	8 × 2 h + 4	12 months	UK	37–66	20	85	100% white
Barnes et al. (2013) [20]	FtF; Individual; CBT	12–18 × 1 h	8 weeks	UK	18–75	26	62	
Beattie et al. (2009) [21]	Remote; Individual; CBT	10	0–2 weeks	UK	20–69	24	71	
Bendelin et al. (2011) [22]	Remote; Individual; CBT	over 8 weeks	6 months	Sweden	20–62	12	42	100% native Swedish
Boggs JM et al. (2014) [23]	Remote; group; MBCT	8 × 1–1.5 h	End-of-treatment	USA	46.89	38	71	89.5% white
Choi et al. (2014) [24]	FtF or Remote; Individual; PST	6	36 weeks	USA	62.43	42	81	52% black/Hispanic Low income homebound older adults
Cramer et al. (2011) [25]	FtF; Group; CBT/PST	12 + 2	1–14 weeks	UK	30–55	20	100	Deprived areas
Fathi et al. (2016) [26]	FtF; Group or Individual; CBT	Group: 17 × 2 h Individual: 17 × 1 h	0, 1 & 6 months	Austria	30–60	23	65	Iranian migrants
Finucane and Mercer (2006) [27]	FtF; Group; MBCT	8	3 months	UK	29–59	13	77	
French et al. (2017) [28]	FtF; Individual; CBT	12–18 × 1 h	Approx 4 years	UK	28–70	20	55	
Gerhards et al. (2011) [29]	Remote; Individual; CBT	8 + 1	0–12 months	Netherlands	43.6	18	50	
Glasman et al. (2004) [16]	FtF; Group or Individual; CBT	6 weeks - 6 years (median 4.5 months)	3–10 months	UK	26–68	9	44	100% white
Glueckauf et al. (2012) [30]	FtF or Remote; Group and Individual; CBT	12 × 1 h (7 group+ 5 individual)	During	USA	58.09	10		African American dementia caregivers
Heilemann et al. (2016) [31]	FtF; Individual; schema therapy	8 × 2 h	3 months	USA	31	8	100	Low income 2nd generation Latinas
Holst et al. (2017) [32]	Remote; Individual; CBT	12 weeks	1–36 months	Sweden	27–68	13	54	
Kahlon et al. (2014) [33]	FtF; Individual; CBT	Unspecified	During	UK	19–54	7		71% white
Knowles et al. (2015) [34]	Remote; Individual; CBT	6–8	4 months	UK	29–69	36	72	94% white
Lillevoll et al. (2013) [35]	FtF and Remote; Individual; CBT	5 online + 7 face-to-face x30mins	End-of-treatment	Norway	22–61	14	64	
Mason and Hargreaves (2001) [36]	FtF; Group; MBCT	Unspecified	0–30 months	UK	24–59	7	71	
Murphy and Lahtinen (2015) [37]	FtF; Group; MBCT	8 × 2 h	3–12 months	UK	41–60	6	83	83% White
Schuling et al. (2018) [38]	FtF; Group; MBCL	8 × 2.5h	End-of-treatment	Netherlands	37–71	17	88	
Smith et al. (2007) [39]	FtF; Group; MBCT	8 × 2 h	0 & 1 year	UK	65–88	38		White British older people
Straarup and Poulsen (2015) [40]	FtF; Individual; CBT or MCT	24 × 50 mins	Unspecified	Denmark	20–35	6	67	
Williams et al. (2018) [41]	FtF; Group; MBCT	8 × 2 h	0 & 6 months	UK	65–78	13	69	older people
Wong (2011) [42]	FtF; Group; CBT	10 × 2.5 h	End-of-treatment	Hong Kong	36.5	20	80	Chinese

*CBT* cognitive behavioural therapy, *FtF* Face-to-face, *MBCL* mindfulness based compassionate living, *MBCT* mindfulness-based cognitive therapy, *MCT* metacognitive therapy, *PST* problem solving therapy

**Table 2:** Study characteristics (AR approaches)

Study	Therapy	Sessions (n x duration + number of follow-ups)	Data collection (time after end-of-treatment)	Country	Age (range or mean)	Sample size	% female	Key demographics (where available)
Danner et al. (2007) [7]	FtF; Group; narrative therapy based on relational and cultural theory	10–12 × 2 h	2–4 weeks	USA	42.6	14	100	Hmong people
Goldman et al. (2016) [43]	FtF; Individual; counselling	6–20	0–12 months	UK	32–62	12	83	
Hellemans et al. (2011) [44]	FtF; Group; MST	6 × 1.5 h + 1	During	Belgium	44	24		
Leonidaki et al. (2016) [45] and Leonidaki et al. (2018) [46]	FtF; Individual; DIT	16	3–10 months	UK	27–60	5	40	40% White
von Below et al. (2010) [47]	FtF; Individual or Group; PP	7–48 months	End-of-treatment & 1.5 years	Sweden	18–25	17	82	

*DIT* dynamic interpersonal therapy, *FtF* Face-to-face, *MST* Multi-Systemic Therapy, *PP* psychodynamic psychotherapy

**Table 3:** Study characteristics (Mixed AR and CB or other approaches)

Study	Therapy	Sessions (n x duration + number of follow-ups)	Data collection (time after end-of-treatment)	Country	Age (range or mean)	Sample size	% female	Key demographics (where available)
Antoniou, Cooper, Tempier, & Holliday (2017) [48]	FtF; Individual; Pluralistic	20.5 (mean)	1–4 weeks	UK	18–58	18	78	100% white
Dakin and Arean (2013) [49]	FtF; Individual; PST or ST	12	0–9 months	USA	74	22	59	91% white older adults, mild executive dysfunction
Krause et al. (2018) [50]	FtF; Group or Individual; Public sector psychotherapy (various models)	22 (mean)	After completion	Chile/Columbia	21–68	24	79	Chilean/Columbian economically disadvantaged
Lambert D’raven et al. (2015) [51]	FtF; Group; Happiness programme	6 × 2 h	End-of-treatment	Canada	19–79	24		
Mörtl and Von Wietersheim (2008) [52]	FtF; Individual and Group; CBT or PP	8 weeks intensive (3x group and 3x individual per week)	During & 3 months	Germany	18–56	26	73	
Nikendei et al. (2016) [53]	FtF; Group and Individual; Multi-modal inpatient or day clinic	8 weeks intensive	4 weeks	Germany	18–60	35		
Valkonen et al. (2011) [54]	FtF; Individual; LTPP or SFT	9–12 (SFT) 194–378 (LTPP)	0–5 months (SFT) End-of-treatment (LTPP)	Finland	20–44	14	57	

*CBT* Cognitive Behavioural Therapy, *FtF* Face-to-face, *LTPP* long-term psychodynamic psychotherapy, *PST* problem solving therapy, *ST* supportive therapy, *SFT* short-term solution-focused therapy

All relevant author interpretations were coded where they concerned the experience or impact of therapy. Interpretations that related only to pre-therapy experiences of depression or pre-therapy expectations were not included unless they related directly to experiences of therapy in some way. Two authors coded five articles independently and then discussed the process of coding and nature of code labels to ensure an overall consistent approach. They then coded the remaining articles independently. When all articles were fully coded, the first author checked coding of all articles for consistency and undertook merging and renaming of codes for consistency and accuracy, following the principles of thematic analysis [55]. A final list of codes was then sorted into a set overarching themes and sub themes which were then discussed among all three authors and refined.

Using the attribute tool in NVIVO, each study was allocated to a model of therapy. These were categorised broadly as either those focussing on cognitions and behaviours (CB; including CBT, schema therapy, MBCT and solution focused models) or those focusing on attachments and relating (AR; including psychodynamic, narrative, systemic and counselling). Some studies included both CB and AR approaches (see Tables 1, 2 and 3 for study classifications). Therapies were also categorised by delivery style (face-to-face or remote) and a delivery format (group or individual). Crosstab queries were used to assess whether certain codes appeared across all types of therapy or if there were some codes which were specific to certain types of therapy or delivery. Since the therapy models and format were not equally represented, this was not a quantitative frequency exercise but was used only to gain a qualitative sense of the representation of codes among certain models or formats. Frequencies are therefore not presented as this would be misleading, but within each theme we comment qualitatively on any particular issues of representativeness across the set of studies.

## Results

### Study selection

**Fig. 1:**
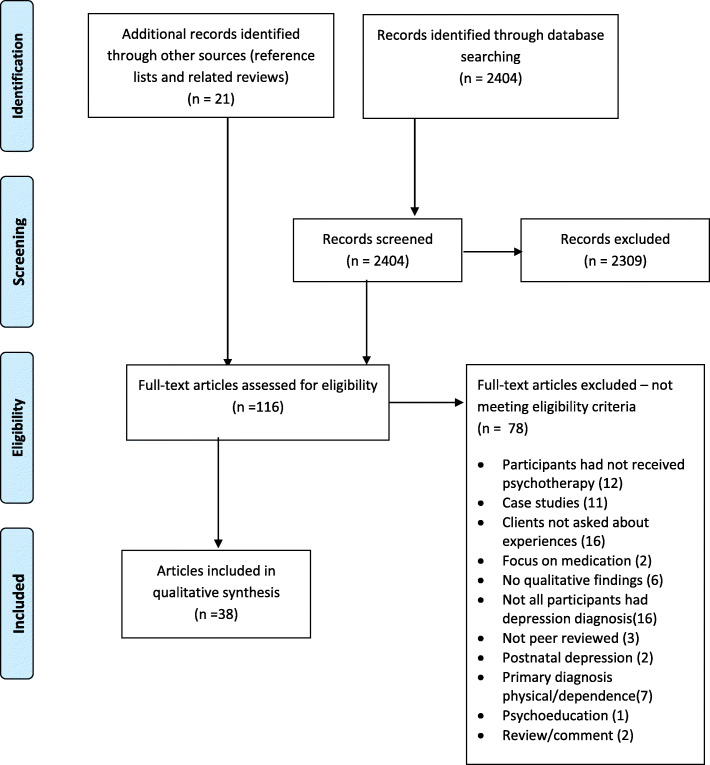
Prisma Flowchart

The initial search identified 2404 studies. A total of 116 articles were reviewed in full, including 21 identified from other sources (reference searches and other reviews). Of these, 38 articles representing 37 studies met the eligibility criteria (see Fig. 1).

### Included studies

Tables 1, 2 and 3 present the key features of the 37 studies included. Studies include a wide range of therapy models delivered in various formats including face-to-face, online and telephone plus group and individual therapies. Studies had been carried out in the UK and Europe as well as the US, South America and Hong Kong. While the majority of participants across the studies were white females (see Tables 1, 2 and 3), there were also a significant proportion of men represented as well as participants from diverse ethnicities including Chinese, Hmong, Black, Hispanic and Iranian. Some studies included participants who were in a position of economic disadvantage. The age of participants ranged from 18 to 88 and the total number of participants represented is 671. Qualitative data were collected at varying time points including during therapy, at end-of-treatment and at various later points up to 4 years post-therapy.

Qualitative methods used among the studies included thematic analysis, grounded theory, framework, template analysis, holistic processing of linguistic complexity, (inductive) content analysis, interpretative phenomenological analysis or a generic phenomenological approach, systemic text condensation, constant comparison and hermeneutic circle approach.

### Quality appraisal

There has been an ongoing discussion on the use of quality criteria for qualitative research in terms of whether articles of poorer quality should be included in meta-syntheses [56]. For the purpose of the current review, all identified articles were included since the eligibility criteria for inclusion in the review ensured that all included studies already met required parameters of suitability of method and design. The only element of the Critical Appraisal Skills Programme (CASP) tool where there was more variability in quality was the rigour of data analysis in that some studies produced more descriptive or superficial analysis while others were more in depth. Nevertheless, all provided some valuable insights (or ‘findings’) on patients’ experiences and so were included. The relatively small sample sizes characteristic of qualitative research are partly mitigated by combining the samples and findings in metasynthesis. Specifically, while some studies had relatively small homogenous sample sizes in keeping with the chosen methodology, the combined sample of 671 represents relative ethnic, gender and age diversity (see Tables 1, 2 and 3). Further, where authors may have an explicit or implicit bias towards a specific treatment modality, this is partly mitigated in review in that the three current authors are independent of all of the studies and treatment modalities. Specifically concerning potential bias of the current reviewers, the first and second authors are healthcare researchers not trained or practicing in any form of psychological therapy. The third author is a trainee clinical psychologist currently training in a range of different psychological therapies with no particular affiliation to one modality.

Three main themes are detailed below beginning with the role of therapy features and setting; therapy processes and how they impact on outcomes; and therapy outcomes (benefits and limitations). Sub themes are reported within these main themes below. As noted above, all quotes provided to illustrate the themes are author interpretations - no primary data is presented.

### The role of therapy features and setting

In addition to the therapy model and delivery format and style, study findings suggested that a range of other features of therapy may impact on patients’ experiences. Specifically, aspects of the therapy environment and structure could impact in certain ways. For example, in a day treatment programme which provided a mixture of group and face-to-face therapies on an intensive basis, participants appeared to feel they benefitted significantly from a regular daily routine.

*The possibility of being able to spend all day in the unit and the regular daily routine was seen as particularly positive and was reported to facilitate personal activity* [53].

In group formats, it appeared that the group size could impact on the usefulness of the group with smaller groups facilitating discussion better.

*Some felt it was easier to concentrate and more relaxing when the group was split into two for specific discussions* [41].

Across all types of therapy, delivery format and style, place and environment appeared to be important. This ranged from issues around privacy of the clinic setting and physical spaces, the peace and tranquillity of the therapy room to physical access issues including transport which could create more or less stress prior to arriving at therapy.

In the DIT studies, the time limited nature of the intervention relative to traditional psychodynamic psychotherapy appeared to be experienced as useful to help with focusing on the most relevant or important issues but also appeared to generate anxiety around the brevity of time and the wish to get as much out of the sessions as possible [45].

In a number of studies of online individual CB approaches, participants were reported as expressing willingness to try novel approaches and technologies and this was reported to be a factor in participants’ experiences of the online format.

*In comparison to traditional remedies participants reported treatment as a new, exciting and better way of receiving care* [22].

Another key element of therapy features which could impact on experience related to accessibility of therapy versus regularity. This is particularly relevant to considering the benefits of remote therapies versus face-to-face therapies. The enhanced accessibility of remote therapy appeared to be highly valued by participants receiving individual online CB therapy. However, in contrast, frustration with technological difficulties was a common finding in studies of individual online therapies.

*Someone thought that the technology worked perfectly while most had some kind of technical problems …*. *Several described the importance of the smoothly working technology since depression means low tolerance for adversity* [32].

As a contrast to this, having regular reliable contact with a therapist face-to-face appeared to be valued, for example in individual psychodynamic therapy and counselling in which the consistent timing of the therapy slots may be part of the theory of change within the model.

*The counsellor is seen as a consistent and containing support with whom clients can feel safe* [43].

There were a number of ways in which the therapy setting or features appeared to either exacerbate or help prevent patients feeling stigmatised by their participation. The nature or branding of an intervention, for example, was reported to reduce the felt sense of stigma. The ‘Happiness programme’ [51] is an example of how simple labelling could destigmatise an intervention. Studies of face-to-face individual CB therapies found that people experienced a sense of stigma from engaging in therapy whereas those receiving remote individual CB therapy appeared to feel that their privacy was enhanced and stigma therefore reduced. At the same time this only applied where individuals had a private computer they could use and the requirement for an internet linked computer could limit privacy if for example it was necessary to use a computer in a more public or even family space.

*The minority who accessed online CBT in other settings (*e.g. *used a relative’s computer) found it harder to engage with the therapy, due to concerns about privacy and being interrupted* [21].

### Therapy processes and how they impact on outcomes

#### Therapy processes: what works

A common feature of all therapeutic modalities and modes of delivery was that therapy appeared to help through certain processes including through enabling sharing and talking about feelings to others (in a group or just to the therapist); and providing a valuable space to talk about one’s feelings. A key process commonly referred to was of participants gaining new insights to help reframe emotions, problems or to increase awareness and also helping to identify existing coping mechanisms. New insights were applied in order to manage feelings and also later on after therapy to help prevent relapse.

*..treatment had encouraged them to revise their perceptions of depression and of themselves. They seemed to have gained a greater understanding of themselves and their current situation by working with the material and expressed that they had acquired specific insights to help them cope with their depression* [22].

*Women explained that the sessions helped show how the patterns from childhood were still setting them up for misery in their relationships today. This was strengthened by the ability to recognize their emotions and that, rather than staying the same forever, negative feelings do pass. This gave them a valuable sense of hope and assurance* [31].

*The meaning of the symptoms changed. They did not threaten her sense of self anymore, because she could understand them better* [54].

Face-to-face group therapies appeared to have some specific features that were useful. Group membership was reported to facilitate upwards or downwards comparison with others, helping to put one’s own difficulties in perspective; group membership was also reported to provide social support and groups were reported to provide a sense of comfort through sharing similar experiences.

*For several people this seemed to be an important normalising process. Themes such as being understood by the group, realising that you were not alone and being able to show emotion in a safe environment, emerged as common positive aspect to being in a group* [27].

In studies on group interventions, studies found that it was important to be able to trust other group members. Groups could also be useful through members learning how others apply certain techniques.

A narrative group for Hmong people based on cultural theory appeared to be particularly valued for the additional social activities involved [57]; while multi-systemic family groups were reportedly valued for their involvement of one’s own family in treatment.

*They experienced the self-disclosure of their own family members, in particular of their children, or of other group members as helpful … They also reported that it was helpful that their family members were able to discuss different issues … patients benefited that their children gained a better understanding of the depression* [44].

Across both CB and AR approaches, studies found that behavioural changes inspired by therapy were felt to directly activate improvements in mood; and that take-home materials of a wide variety were felt to be useful for ongoing work between therapy sessions. While this finding dominated studies of CB approaches in which take-home materials are a common feature, this also applied for example to DIT in reference to the ‘end-of-therapy’ letter which ‘helped the digestion of the therapeutic material after therapy had ended’ [45]. In individual CB approaches, in-between session work appeared to be experienced as a valuable part of the therapeutic process. In face-to-face CB approaches, studies found that the techniques for managing feelings could become naturalized and automatic and this was a key part of the process.

*It was only when these individuals were asked how they managed a “low day” that it was evident that they were unconsciously or automatically using CBT skills* [28].

Across all face-to-face types of therapy, learning the importance of focusing on one’s self was described as a key process. All face-to-face individual therapies found that participants experienced being enabled to process the past. Likewise individual therapies seemed to enable people to learn to reflect on their problems. Face-to-face therapies were reported to help through impacting on causal attributions about depression.

*This was achieved through a reconceptualisation of the aetiology of their problems, which in many cases involves the convergence of factors belonging to multiple areas* [50].

#### Therapy processes: what’s unhelpful

This sub-theme concerns aspects of therapy that were seen as unhelpful, which varied by type of therapy. Across CB and AR approaches, studies found that therapy could emphasise or confirm problems and that some participants could experience interaction with the therapist as difficult; sometimes participants reported blaming themselves for non-improvement. In group approaches, there were findings suggesting that too much time was sometimes spent sharing instead of focusing on issues.

Unhelpful aspects specific to CB approaches included doubting the usefulness of homework and finding the initial formulation too difficult to understand. With online CBT (therapy by email), it was found that typed exchanges between patients and the therapist could be felt as awkward and cause unwelcome time delays. In face-to-face group CB therapies, group members’ negative emotions could be experienced as overwhelming.

*A number of participants mentioned that they were overwhelmed by the emotions displayed by others in the group, particularly in the early stage of the group process* [42].

The requirement to practice or do homework in CB approaches was sometimes found to be burdensome; participants could feel overwhelmed by the techniques they had to learn to use; and the volume of material and information could feel excessive.

*Each session brought a new practice and practices were seldom revisited in the curriculum … the number of options to choose from for home practice seemed to be confusing to participants* [38].

Specifically in MBCT, the practice of the “body scan” was reported to trigger flashbacks or severe anxiety for some participants with a history of abuse or trauma [27]. In a study of public psychotherapy for economically disadvantaged Chileans and Colombians, it was found that therapy could sometimes feel unfocused and unclear [50]. Two studies, one with Hmong people and one with older white Americans found that therapy was felt to ignore spirituality [49, 57].

#### Issues integrating therapy with real life

This sub theme concerns the inter-relationship between therapy and ongoing daily life. These issues were raised across all forms of therapy and modes of delivery although appeared much less relevant to AR approaches because most of the issues raised relate mainly to the use of specific techniques taught within CB approaches for managing everyday thoughts and feelings. Several studies across CB approaches found that even where participants found the ideas, concepts and practical aspects of the techniques taught in these therapies useful in principle, they struggled to apply them in real life.

*… the training did not work because it was too ‘artificial’ and too far from real-life issues* [40].

Studies also found that participants reported certain practical barriers to maintaining practice of these techniques such as not having time or a quiet space at home.

*… implementing cognitive strategies as suggested in therapy was simply not practicable given the time and circumstances. Sarah, for example, sometimes felt she did not have the quiet and space to ‘stop and think’ because she lived in cramped accommodation with her mother* [16].

Some findings did however indicate that some participants felt able to overcome these barriers.

*One woman felt that making the time to practice the longer meditation was ‘too much of a luxury’ when she had 6 children at home and instead practiced mindfulness of washing the dishes and mindful walking … Others adopted a more flexible attitude towards practice* [27].

Specific to face-to-face CB approaches, it was reported that some participants were able to build some of the techniques they had learnt into their life and lifestyle and were then able to benefit from them.

*She was doing a lot of informal practice, for example while driving, chopping vegetables, or gardening* [39].

Although most standard therapies do not provide for the therapist to get involved with the clients’ problems of living, thus creating a separation of social and psychological issues, one study of an AR approach found that among Hmong women living in the USA, therapists providing practical support with issues of living was seen as key to establishing a therapeutic relationship.

*Group facilitators, acting as advocates in these areas and helping the women to connect to useful resources, seemed to increase the legitimacy and trust the women placed in the group therapy experience* [57].

#### Patient preferences and individual difference

This sub theme concerns patient preferences for certain styles of therapy and individual differences which impact on the experience of therapy. Across all modes of therapy was the finding that therapy expectations can impact on therapy outcome in a number of ways. For example, people with positive expectations might be more motivated to work in therapy.

*They also explained how their increased expectation of treatments made them completely motivated to be more active and productive during the interventions* [26].

Among CB approaches, findings suggested that choice of therapeutic model and content should be based on the individual or tailored to their needs. It was also claimed that patient preference could impact on engagement and therapy satisfaction in CB approaches.

*… those patients who reported negative views of cCBT [computerised CBT] said they deliberately did not use the programme after the initial attempts, indicating poor engagement due to deliberate nonadherence* [34].

One such preference that might be taken account of is whether people wish to focus on issues of the present or the past and this preference might lend itself to different models or approaches within a model. Further, specific to CB approaches, findings suggested that some standardised content is not always felt as relevant to individuals and should be tailored. Across all approaches it appeared that different people may find different aspects and elements helpful. For example, people might prefer different techniques to others, some people might appreciate online communication and others might find it frustrating. Some studies found individual differences so fundamental that they grouped participants. For example a study on remote CBT [22] grouped participants into ‘strivers’, ‘readers’ and ‘doers’ reflecting their different approaches to interacting with the online programme.

It was also reported that people’s expectations around therapy could influence their levels of engagement in CB approaches and therefore have a positive or negative impact. Some studies found that there were varying degrees of willingness among participants to invest time in practicing CB techniques and this impacted on therapy effectiveness. Techniques and tools were also often adapted by individuals to suit their lifestyle or way of doing things.

*It was evident that the proposed approaches and techniques were not universally suitable, and some patients went to considerable lengths to restructure the content to suit their perceived needs* [35].

#### Challenges of undertaking therapy

This sub theme concerns findings across several studies which indicate the sorts of challenges people appear to experience when undertaking therapy. Confronting painful emotions in therapy was reported to be a particular challenge across all therapy models and delivery formats.

*During the course of their treatment, they had discovered aspects of themselves which they felt unhappy about – for example, realizing that they had social anxiety … or had needed to revisit difficult periods of their lives and thus painful memories* [20].

It was also found that people could experience talking about one’s self and sharing emotions as very difficult and this was common across face-to-face therapy model.

*Group psychotherapy patients typically experienced difficulties sharing their problems with the group. They could see their own problems as minor or ‘censor themselves,’ as not everything seemed permissible* [47].

Studies found that although people might recognise the need to change their thinking or behaviour, this was felt to be very hard to do. Again this was common to all main face-to-face models.

*Participants described not being able to break familiar well-rehearsed patterns and responses with family members. Terry talked about always in the past finding his family too smart for him, and even now when he went home he was immediately drawn into the familiar role of being ‘pushed aside’* [16].

Nevertheless, some studies found that participants recognised the benefits of working through challenging aspects of therapy and some studies also found that people were prepared to make more effort even when they found that they were not yet seeing any improvements resulting from therapy.

*Failing to improve from treatment made participants express a wish of going back and working more with the material hoping to profit from it* [22].

#### Importance of the therapist

The importance of the therapist to the experience of therapy was a key theme across many studies. Some aspects of this theme were specific to the difference between remote and face-to-face therapy. In remote CBT for example, lack of therapist support was found to relate to dropout or poor engagement.

*The perception of not being taken seriously, as being one in a pile of depressed individuals offered a panacea, led to little confidence in iCBT [internet CBT]* [32].

It was also found that in remote CB approaches, genuine interaction was felt to be a key missing ingredient, that it was not possible to “establish a meaningful therapeutic relationship online” [21] and that the lack of therapist support was felt to reduce the impact of therapy.

To some extent this limitation could be mitigated through interactive or live support aspects and it was found that these elements were essential to mitigate the lack of a therapist.

*The relationship with the therapist had a function beyond supporting MoodGYM use, in providing an arena for sharing thoughts and feelings and receiving feedback and advice* [35].

There remained a strong sense from study findings that face-to-face interaction with a therapist could improve the experience of therapy and patient satisfaction. Thus, face-to-face therapy was depicted as a collaboration between the patient and therapist and the therapist was described as key to facilitating new insights and skills development. In specific cross-cultural settings, the therapist also appeared to be seen as being able to facilitate cultural adaptation to the nature of therapy given that the individual problem focus of Western therapy does not naturally fit with some other cultural perspectives on emotional problems. Given the central role of the therapist, a good relationship with the therapist was found to be particularly important to patients in individual therapy. It was also reported as being important to participants that they felt understood by their therapist and that the therapist displayed empathy and other positive characteristics such as being accepting, non-judgemental, reassuring, normalising, caring, respectful, soothing, calming, professional, kind, warm and compassionate. In particular, studies of individual face-to-face therapy found people placed high value on the therapist listening, understanding and working collaboratively.

*… there was a sense of the counsellor listening with understanding. This was felt to be purposeful listening to ascertain clients’ emotions and the personal meanings attributed to what they were discussing in their sessions* [43].

### Therapy outcomes (benefits and limitations)

#### Benefits of therapy

A range of benefits of therapy were identified which featured across all therapy models, formats and delivery types. These were that therapy was reported to improve symptoms; enable people to change or improve their way of relating to others; and empower people. An additional benefit which featured in the majority of studies was that therapy was felt to enable participants to use techniques learnt in therapy to manage their everyday thoughts and feelings.

*… participants spoke of using this strategy during stressful situations such as entering a crowded room. In the accounts of half the participants intentionally refocusing attention was associated with a positive impact on depression-related mood and thinking* [19].

Further benefits found across all face-to-face models were that therapy was felt to enable lifestyle or behaviour changes; and improve self-knowledge, self-belief or self-acceptance.

*Participants reported rediscovering lost identities and realized that difficult feelings pass. Additionally, participants were able to think more clearly and become more confident and self-accepting* [33].

CB approaches of varying formats were found to provide techniques to help prevent relapse. Specific to face-to-face CB models, therapy was found to help break habitual thinking or behaviours; and specific to individual face-to-face CB approaches, therapy was reported to benefit even if people had negative expectations or experiences. In a study of PST for older people, therapy was found to also have an impact on participants’ memory [49].

#### Limits to therapy

This sub theme concerns the sense that whatever value or benefits therapy can bring, there are inevitably limits to what can be achieved in therapy. In particular, findings indicated that therapy irrespective of model, was often reported to leave family, social or health problems unresolved, perhaps through the focus on individual psychological problems and the disconnect people often feel between therapy and situations they face in real life noted earlier.

*Interviewees saw their problems as being related to their situational conditions, for instance marriage problems, conflicts in a job, interrupted studies or economical difficulties. Even though they felt that psychotherapy and the therapist were giving valuable support in a difficult situation, they felt that solutions were not found for the acute problems* [54].

Studies also found that participants often considered therapy as only one part of a wider process required to live a better life and that therapy was not felt to have all the answers.

*Participants viewed CBT as part of a process of self-improvement that began before therapy and contained other change elements, like giving up smoking … and drinking* [16].

Specifically relating to individual face-to-face CB approaches, it was found that people could be left feeling as though they had not had the chance to explore the underlying causes of their depression properly. This and other factors meant that people appeared to be left feeling they had missed out on potential benefits from therapy and a common finding was that participants expressed a wish for more therapy or a different type of therapy.

*While most of the group found the course enjoyable the majority of the group thought the course was too short* [27].

*The lack of depth could be perceived as due to lack of time because of finite sessions, or as something fundamental to the model of CBT itself* [20].

#### After therapy ends

This sub theme describes a range of findings relating to how people feel after therapy has ended. Therapy was reported to provide a number of benefits during and after therapy, described earlier. Such positive experiences, particularly in group CB approaches, were found to encourage ongoing practice after ending. However, studies also reported a range of negative experiences after therapy had ended. Some CB studies found that participants reported losing motivation to practice techniques once therapy had ended.

*These individuals mentioned that they tended to repeat the techniques they had learned from the interventions, but as soon the interventions ended they lost their motivation* [26].

In all therapy models, it was found that ending therapy could be experienced as a loss.

*This group also described the difficulty of treatment ending, with many participants in the group described feeling lost, suggesting that they still continued to rely on their therapist for help in the management of their depression and had not gained the same sense of self-dependency or sense of control* [28].

Similarly, two studies both involving group face-to-face therapy [25, 53] found that people could find themselves feeling more alone after therapy had ended.

*Some patients reported feelings of loneliness after discharge as the close relationships with the unit’s other patients had ceased with the end of treatment* [53].

Related to the above indications that ending therapy led to loss of motivation, a sense of loss and feeling more alone, it appeared that irrespective of therapy modality, symptoms were reported to persist or relapse among some participants after therapy .

## Discussion

The review findings highlight a range of ways in which certain types or formats of psychotherapies can be experienced by people with depression in terms of the processes that may relate to benefits as well as harms that arise. The findings emphasise that there are a number of important common factors across psychological therapies which can impact on patients’ experiences of therapy and outcomes. The discussion below considers some of the key common factors highlighted in relation to related literature and also considers issues arising in relation to negative effects of psychological therapies.

Some of the factors common to psychological therapies highlighted in this review have been identified in quantitative common factors research such as the importance of setting and the role of the therapist. There is, for example, extensive evidence that the therapeutic relationship is central to therapy process and outcomes [58] and that specifically, therapeutic alliance, the most researched element, has an effect size (Cohen’s d) of 0.58 in meta-analysis [59]. Similarly, a metasynthesis of patient experiences of computerised therapy for anxiety and depression emphasised the role of common factors across different formats of computerised delivery [11]. However, the current review findings emphasise the significant limitations of remote therapies in which key common factors are removed. Specifically, findings illustrate clearly why many people who receive remote therapies in which the therapist is replaced by a computer programme, feel left wanting something more, in the absence of genuine interaction with a therapist. Similar to findings from the above metasynthesis of experiences of computerised therapy, the limitations of remote therapies may be partly mitigated by sensitivity of the remote therapy to individuals’ sense of self, taking into account differing individual needs, preferences and the impact of an individual’s symptoms on their ability to engage with remote delivery.

Less evident from quantitative research is the role of stigma and, specifically, how stigma impacts on willingness to engage in psychological therapy. In particular, the review findings illustrate how, in spite of the limitations around lack of therapist interaction, remote forms of therapy can go some way to addressing stigma through creating the possibility of privacy. Similarly the metasynthesis of patient experiences of computerised therapy for anxiety or depression [11] indicated that it can be empowering to self-tailor therapy delivered in a computerised format; but in the absence of a therapist, this may be burdensome, requiring motivation and self-discipline. The current review suggests that alternative means of reducing stigma are also feasible without necessarily removing the therapist or the physical setting. For example, simple changes such as therapy name and location can reduce the shame of coming forward for help. Regular attendance at a non-stigmatising setting can then reduce shame further by enabling face-to-face sharing of experience and information with others.

Also less evident from quantitative research is the limited relevance of psychological models and techniques to individual life contexts and their inability to help clients with their immediate family or social problems that may be triggering or maintaining depressive experiences. This finding came up often among several forms of therapy. It is not a finding that would be identified within most psychological therapy trials because the focus tends to be on symptom outcomes. In an overview of targets and outcomes in psychotherapy research [60], only one meta-analysis was identified which reviewed quality of life outcomes in trials of psychotherapy for depression. The meta-analysis referred to [61] found only 44 RCTs of psychotherapy for depression (a minority of depression RCTs published) reporting a quality of life or functioning outcome. Moreover, while some quality of life measures may ask about issues relating to social support and relationships, many focus on individual functioning or health and may not reflect patient priorities [62]. The findings in the present review concerning social and cultural contexts lend support to calls for a greater focus on non-symptom outcomes and specifically for outcomes to be patient-focused and patient defined: “Patients are the ones who suffer from mental disorders and, as long as we do not exactly know what these disorders are or what their causes may be, we should rely on the ones who suffer from them to decide what outcomes should have the priority” [60]. This may also then lead to greater recognition of the extent to which psychological therapies have become defined by individualistic Western epistemologies which place responsibility on patients to change their ways of thinking and doing with little consideration of the social contexts in which these changes are expected to take place [63].

The studies in this review which focus on specific ethnic groups or economically disadvantaged groups highlight this particularly well, in that it where the therapy model stepped outside of the traditional boundary and enabled therapists to help with practical issues, this was felt by participants to help enhance the therapeutic relationship and subsequently improve outcomes. Review findings also suggest that group psychotherapies have the potential to cross some of the bridges between individual psychology and social factors. Findings indicated that factors common to groups such as the social element, opportunity to relate, share and compare experiences with others cut across models and enable social development. In some cases this also provided direct social connection where groups could cross the therapeutic boundary and undertake social activities together.

The findings of the present metasynthesis also highlight the importance of considering adverse effects of psychological therapies. These have historically been under-researched in the field and although there has been increasing recognition of the potential for and incidence of adverse effects, they remain under-assessed in clinical trials [64]. While it might be inevitable that some individuals experience therapy as challenging or even unhelpful, there are also findings that reveal the potential for harm, such as reports of finding other group members’ emotions overwhelming; finding practicing certain techniques overwhelming; or finding that the Bodyscan can generate flashbacks. The finding concerning groups is in line with a review of adverse outcomes in group psychotherapies which set out a range of therapist and patient factors that could precipitate negative effects. This review noted that “highly charged” disclosures from some group members can be experienced as overwhelming by other group members and that this may happen in the early stages of a group before it has developed into a “supportive unit” [65]. In spite of this review published two decades ago, there remains limited development in methodologies to monitor such negative effects. A recent review of instruments to monitor negative effects found nine available instruments including one specifically for group psychotherapy published in German [64]. The authors found these measures to be inadequately validated and recommended that work was needed to develop a comprehensive consensus framework which could be used in routine outcome monitoring and research. The findings of the present metasynthesis support the call for such measures but it is also important to highlight that the parameters of any measure should not be designed by researchers alone but should also take into account the first person perspectives of patients which may highlight previously unseen harms or illustrate the ways in which certain processes can lead to harm for some individuals.

### Limitations

Although the review presented provides a comprehensive look at the literature in this field and includes a large number of studies, it is also important to acknowledge that some therapies are under-represented or absent while other therapies dominate i.e. CBT and MBCT. It is important not to conflate absence of knowledge with knowledge of absence which the paradigm of evidence based practice has a tendency towards, so that particular treatments are assumed to be ineffective because of an absence of research rather than research showing negative findings. It is likely that a wider range of experiences have been found among the participants of the CBT and MBCT studies merely because there are more studies of these treatments. Many of the experiences identified in these studies may apply equally to other forms of therapy without yet having data to support this. Where certain findings by their nature appear to relate to specific elements of branded therapies (e.g. Bodyscan), or specifically to group, individual, remote or face-to-face formats, these findings may be considered to reliably differentiate. However, where findings do not have a high degree of specificity to a particular model or delivery mode, caution is required around whether the findings have specificity to the model within which the finding arose.

Another limitation concerns diversity. As noted earlier, by combining the studies, a relatively good degree of diversity is represented among the combined population of participants. However, the non-White populations represented are fairly specific: Iranians, Chinese, African Americans, Chileans, Columbians and Hmong women. Because many of these studies are specifically addressing cultural and ethnic issues, they can be particularly informative about the role of culture in more depth than is possible within RCTs. The range of ethnicities and cultural groups remains relatively limited however. In terms of informing guideline development, NICE, for example, explicitly sought to give special consideration to people from black and minority ethnic groups [66]. While the findings from this review could be used towards this aim, it would be useful if future research exploring patient experiences of psychological treatments for depression ensured greater representation of UK black and ethnic minority and other underrepresented UK populations in order to inform NICE guidelines.

Another limitation is the diversity of ways in which studies classified depression for purposes of eligibility. The review excluded studies in which participants self-diagnosed depression thereby ensuring some formal criteria were met. While referrer diagnosis (usually done by primary care professionals), formal psychiatric diagnostic interviews and self-report questionnaires have been designed or developed with a view to being equally reliable, there are inevitable differences in degree of reliability between these forms of diagnosis in practice.

## Conclusions

Meta-analyses of psychological therapies for depression indicate that several forms of psychotherapy can be effective for reducing symptoms. However, as Cuijpers argues, much of this evidence is potentially subject to publication bias, poor quality, researcher allegiance, lack of long-term follow-up and overestimated effects [67]. Moreover, since trial outcomes tend to be determined by the interests of researchers, practitioners, employers, insurance companies or policymakers rather than patients, it is critical to understand in more depth what therapies are like for those who receive them and to use these findings to improve practice and delivery of therapies. There are significant limitations of psychological therapies as currently configured in UK, US and Europe, particularly in that they are often offered on a short term basis only, that they tend to be restricted to certain theoretical models and that their outcomes have been assessed only in terms of symptoms rather than patient preferred outcomes. It would benefit the field to look at the sorts of cross-model issues identified in the findings in this review in order to improve therapy impact and to include more orientation to social factors.

Findings of this review emphasise that not only should patients be more fully involved in discussions and decisions about which therapies they might be offered, but also in conversations about tailoring therapy to individual needs both before and during therapy. These sorts of discussions should be informed by much clearer information about therapy types and formats and it would be useful for a co-produced patient guideline to be developed which provides clear information based on evidence which might enable patients to make informed choices and engage on equal terms in discussions with their referrer. Therapy providers could also consider taking a more patient centred approach to developing their models, taking into account common factors which patients regard as important, engaging with the well-established field of research which indicates that these are significant predictors of outcome [58].

## Data Availability

Data sharing is not applicable to this article as no datasets were generated or analysed during the current study. The current study drew on and reviewed data already published; all published studies drawn on are cited in the Table and References.

## Abbreviations

AR: Therapies focusing on attachments and relating
BA: Behavioural Activation
CASP: Critical Appraisal Skills Programme
CB: Therapies focusing on cognitions and behaviour
CBT: Cognitive Behavioural Therapy
DIT: Dynamic Interpersonal Therapy
EPA: European Psychiatric Association
EU: European Union
IPT: Interpersonal Therapy
LTPP: Long-term Psychodynamic Psychotherapy
MBCL: Mindfulness Based Compassionate Living
MBCT: Mindfulness Based Cognitive Therapy
MCT: Metacognitive Therapy
NICE: National Institute of Clinical and Health and Care Excellence
PP: Psychodynamic Psychotherapy
PST: Problem Solving Therapy
RCT: Randomised Controlled Trial
SFT: Short-term Solution-Focused Therapy
ST: Supportive Therapy
STPP: Short Term Psychoanalytic Psychotherapy
UK: United Kingdom
USA: United States of America
WHO: World Health Organisation
